# Incidence and Distribution of New Renal Cell Carcinoma Cases: 27-Year Trends from a Statewide Cancer Registry

**DOI:** 10.15586/jkcvhl.v9i2.219

**Published:** 2022-04-18

**Authors:** Ahmad N. Alzubaidi, Stephen Sekoulopoulos, Jonathan Pham, Vonn Walter, Jay G. Fuletra, Jay D. Raman

**Affiliations:** 1 Department of Urology, Penn State Health Milton S. Hershey Medical Center, Hershey, PA, USA;; 2 Penn State College of Medicine, Penn State Health Milton S. Hershey Medical Center, Hershey, PA, USA;; 3 Department of Public Health Sciences, Penn State University College of Medicine, Hershey, PA, USA

**Keywords:** diagnosis, kidney cancer, renal cell carcinoma, statewide

## Abstract

Nationwide databases have implicated an increased incidence of renal cell carcinoma (RCC). The Pennsylvania (PA) Cancer Registry was queried to better define incidence, geographic distribution, and statewide trends of new RCC cases over a 27-year period. JoinPoint Trend Analysis Software modeled average annual percent changes (APCs) in age-adjusted rates (AAR). Maps plotting county-level incidence rates and stage distribution of disease across the state in 5-year time intervals were created using R 4.0.2 software. Overall, 59,628 cases of RCC were recorded in PA from 1990 to 2017. Eighty six percent of patients were >50 years of age, 61% were males, and 89% were Caucasian. Stage distribution using the SEER staging system included 64% local, 17% regional, and 16% distant. Over the study interval, AAR of all RCC cases increased from 9.9 to 18.0 patients per 100,000 population with an APC of 2.3% (p < 0.01). AAR of local disease increased from 5.4 to 12.7 patients per 100,000 population with an APC of 3.2% (p < 0.01). AAR of regional disease also increased from 1.9 to 2.9 patients per 100,000 population with an APC of 1.0% (p = 0.01). Younger patients (<50 years) had a greater rate of increase than older counterparts (APC 3.8% vs. 2.0%, p < 0.05). Geospatial investigation of new RCC cases noted certain geographic concentrations of greater disease incidence. The incidence of RCC in PA has increased over the past 27 years in PA. One-third of the cases are regional or metastatic at presentation and rates of increase were most notable in younger patients.

## Introduction

Renal cell carcinoma (RCC) accounts for approximately 4% of all adult malignancies with nearly 270,000 new cases diagnosed annually worldwide (1). In 2010, 58,000 new cases of RCC were diagnosed in the United States alone, with approximately 13,000 deaths (2). These numbers have increased to an estimated 76,080 new cases and 13,780 deaths in 2021 (3). Specifically, in Pennsylvania, the incidence rate of RCC is 16.2 per 100,000 population with a total of 2700 cases reported in 2018 (4). Previous studies have highlighted that despite an increase in newly diagnosed RCC cases, mortality rates have decreased (5, 6). These observations are likely related to stage migration from enhanced imaging as well as improvements in treatment protocols.

RCC originates from the renal pelvis and is the most common primary renal neoplasm. RCC commonly occurs between the sixth and eighth decade of life and is twice as common to occur in males than in females (5). Numerous different subtypes of tumor exist, each with a unique molecular mutation and clinical prognosis (7). The most common subtype is clear cell RCC, which represents 70–80% of all renal cancers and is regarded as the most aggressive subtype due its intrinsic high rates of metastasis and mortality (6).

Cigarette smoking and obesity are both well-established, modifiable risk factors for RCC. There has been an observed decline in cigarette smoking over the past several decades; however, recurrent toxin exposure takes years to manifest. Thus, while smoking rates are declining, their effects are only now being revealed as the population continues to age. Specifically for obesity, the relative risk of developing RCC is directly correlated to an individual’s BMI (6). Despite an observed decline in the prevalence of cigarette smoking, it is possible that the continued rise in RCC cases could be a result of increased obesity rates (8). Additional factors, such as hypertension, acquired and genetic kidney diseases, and environmental exposures, have been implicated in RCC, but these mechanisms are less understood.

Herein, we have reviewed 27 years of RCC data within Pennsylvania to better understand incidence and trends for RCC, staging of these cancers, and identify geographic “hot spots” with higher rates of disease.

## Patients and Methods

Using the Pennsylvania Cancer Registry, age-adjusted renal cancer incidence rates per 100,000 population between 1993 and 2017 were extracted through the Enterprise Data Dissemination Informatics Exchange (9). Since data were obtained from this publicly available database, this study was IRB exempt, and no informed consent was obtained. We used SEER site recodes to restrict our analysis to patients with Kidney and Renal Pelvis, which included International Classification of Diseases of Oncology, Third Edition/World Health Organization (ICDO-3/WHO) site codes of C649 and C659, excluding histology codes of 9050-9055, 9140, 9590-9992 (lymphomas, Kaposi sarcoma, and mesothelioma). Traditionally, Surveillance, Epidemiology, and End Results SEER database combined Kidney and Renal Pelvis tumors (10). Upper tract urothelial carcinoma (UTUC) is a relatively rare tumor, and includes carcinoma of the renal pelvis and carcinoma of the ureter, which account for approximately 5–10% of urothelial carcinomas (UCs) (11, 12). Given the low incidence of renal pelvis tumors, it is less likely that the inclusion of such a group with kidney tumors will have a significant impact on trends.

The JoinPoint Trend Analysis software (13) was then used to model annual percent changes (APCs) in age-adjusted rates (AAR). This software examines trends of gender, race, and stage through a linear approach that fits incidence rates for multiple years while standardizing incidence rates with the standard population. Maps plotting county-level incidence rates of total and distant disease across the state in 5-year time intervals were created using R software.

The stage distribution for renal cancer at the time of diagnosis was based on SEER Summary Staging system (in situ, local, regional, and distant) as defined by the PA Cancer Registry. In this system, the cancer was staged as in situ when it has not spread beyond the basement membrane of the epithelial tissue involved; local when the cancer is confined to the organ of origin; regional when the cancer extends beyond the original organ to nearby lymph nodes, organs, or tissues; and distant when the cancer extends to distant organs or lymph nodes. As such, the TNM system for classifying renal cancer is not used in this dataset.

## Results

From 1990 to 2017, 59,628 cases of RCC were recorded in the state of Pennsylvania. Descriptive analysis of age, gender, and stage are shown in Table 1.

**Table 1:** Characteristics of patients diagnosed with renal cell carcinoma in the Pennsylvania Cancer Registry from 1990 to 2017.

Variables	Number of cases (Percent)
**Total cases**	59,628
**Age** 0–50 50+	8085 (13.6%) 51,543 (86.4%)
**Gender** Female Male	23,170 (38.9%) 36,457 (61.1%)
**Race** Caucasian Black	52,959 (88.8%) 5659 (9.5%)
**Seer stage** In Situ Local Regional Distant	975 (1.6%) 37,264 (62.5%) 9887 (16.6%) 9439 (15.8%)

**Figure 1:**
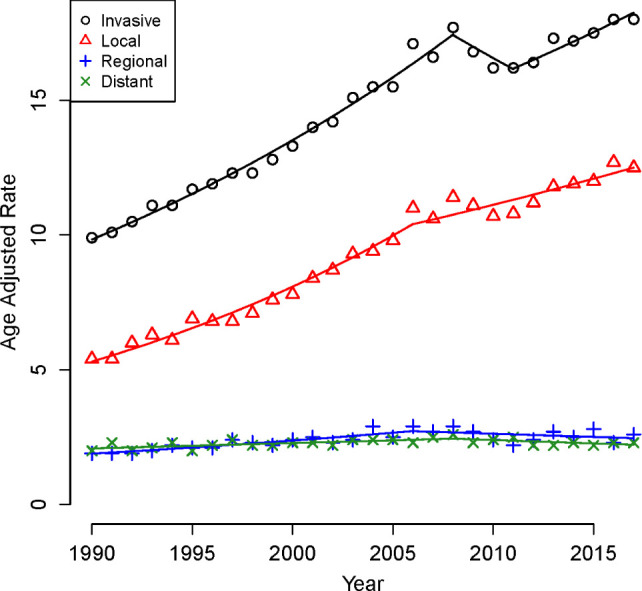
Stage-stratified, age-adjusted rates of RCC per 100,000 population, 1990–2017.

Figure 1 demonstrates AARs of all RCC cases stratified by stage. Overall, rates of RCC cases over the study interval increased from 9.9 patients to 18.0 patients per 100,000 population with an APC of 2.32 (95% CI = 1.74, 2.90, p < 0.01). This increase was primarily driven by increases in patients with local and regional disease. For patients with local disease, AARs increased from 5.4 to 12.7 patients per 100,000 population with an APC of 3.23 (95% CI = 2.93, 3.53, p < 0.01). Similarly, AARs of regional staged disease increased from 1.9 to 2.9 patients per 100,000 population with an APC of 1.00 (95% CI = 0.29–1.71, p = 0.01). Conversely, AARs of patients with distant staged disease were stable, from 2.0 to 2.6 patients per 100,000 population with an APC of 0.23 (95% CI = 0.24–0.70, p = 0.34).

**Figure 2:**
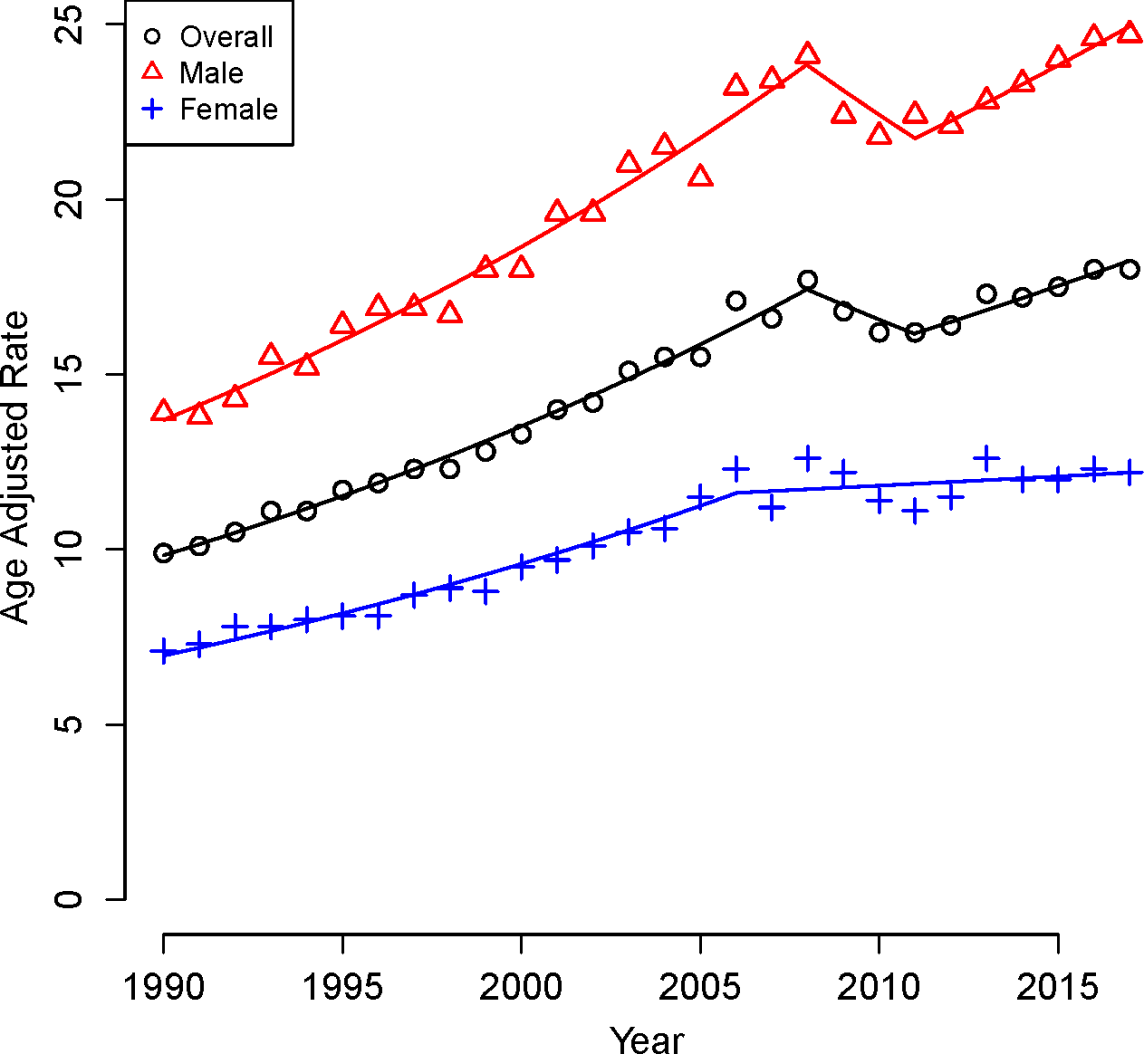
Gender-stratified, age-adjusted rates of RCC per 100,000 population, 1990–2017.

**Figure 3:**
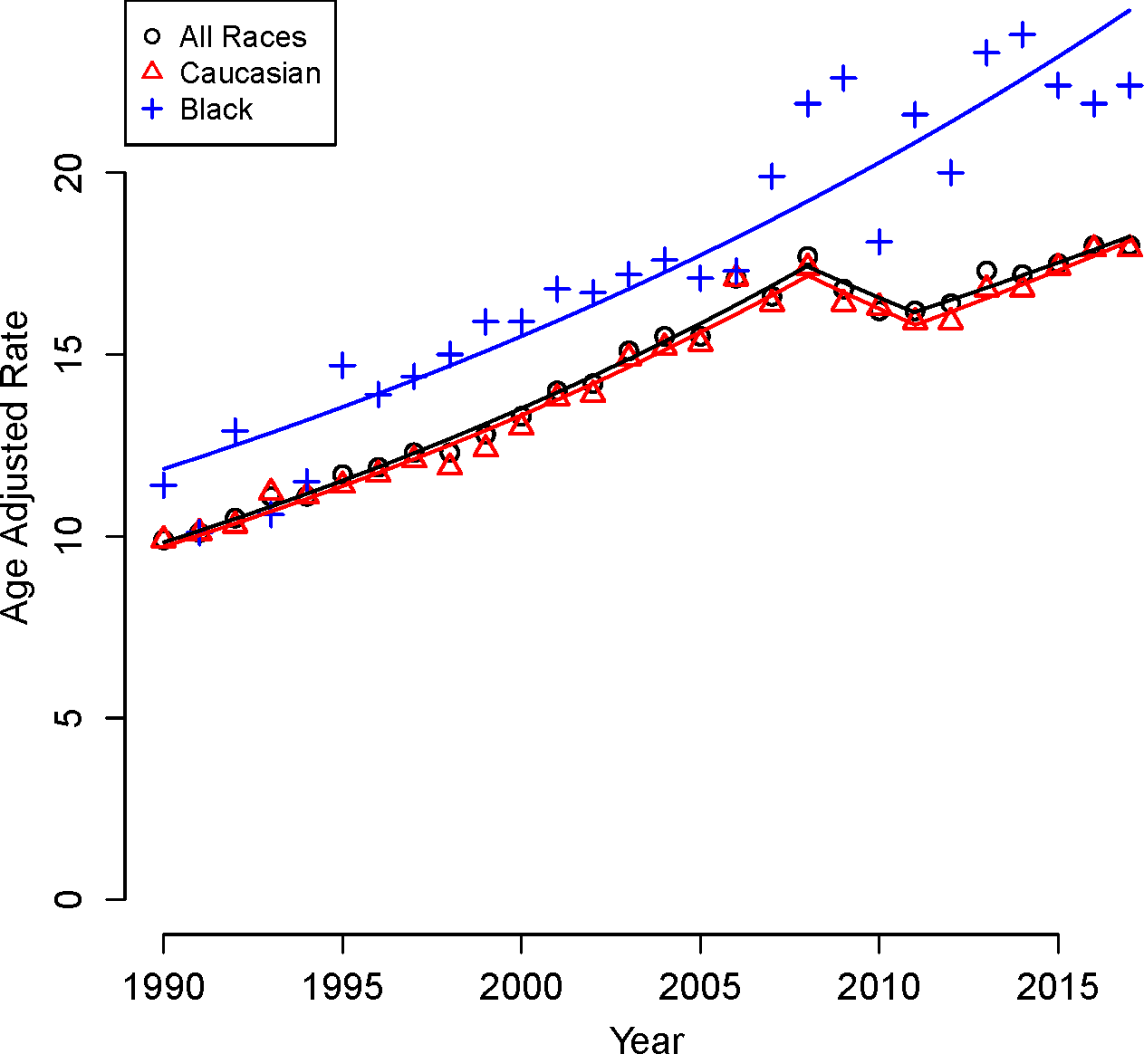
Race-stratified, age-adjusted rates of RCC per 100,000 population, 1990–2017.

Rates of increase of RCC cases also demonstrated differences when stratified by age, gender, and race. Specifically, the APC for younger patients (<50 years) was significantly higher at 3.8% (95% CI = 3.39, 4.17) compared to their older counterparts (APC of 2.0%, 95% CI = 1.58, 2.44, p < 0.05), Figure 1. In addition, rates of increase were greater in males with an APC of 2.25 % (95% CI = 1.49, 3.01) compared to females with an APC of 2.09 % (95% CI = 1.74, 2.44, p < 0.05), Figure 2. Finally, the APC for patients who are of African American race is slightly higher at 2.72 (95% CI = (2.31, 3.14) compared to patients who are of Caucasian race at 2.33 (95% CI = (1.55, 3.12) but did not reach statistical significance p > 0.05, Figure 3.

**Figure 4:**
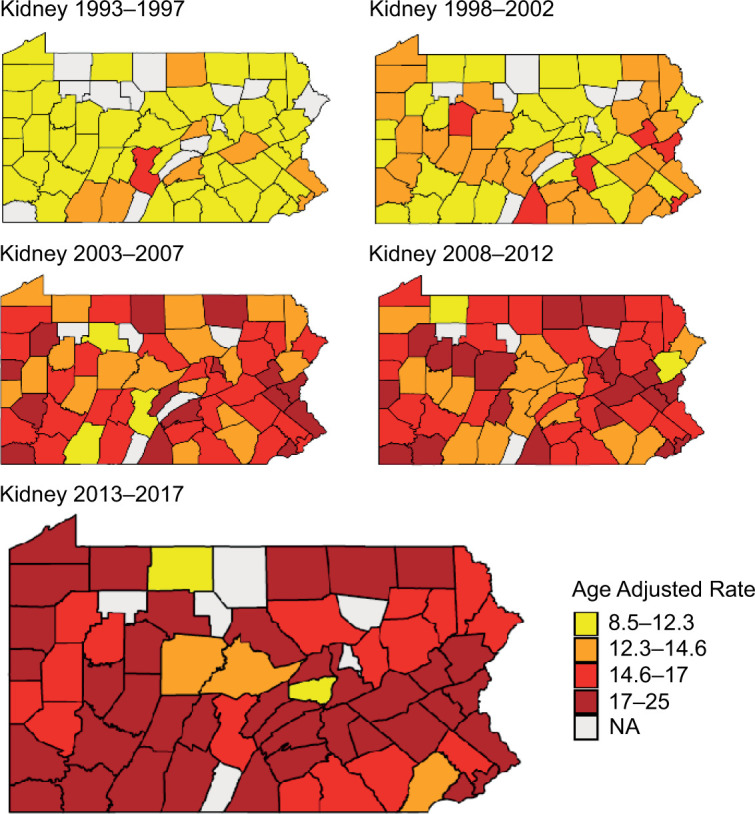
Age-adjusted RCC incidence rates by county in 5-year intervals, 1990–2017.

Figure 4 shows the geospatial investigation of total disease in the state of Pennsylvania delineated into its 67 counties through choropleth maps. Location was based on the home zip code of patients. As noted in the figure, there is a widespread increase in the AAR of RCC with growing hotspots throughout the state from the time periods of 1993–1997 to 2013–2017.

## Discussion

The goal of this study was to analyze data available from a large statewide cancer registry to evaluate the incidence, trends, and geographic distribution of newly diagnosed RCC. We found that approximately 86% of patients with RCC were in the older population (>50 years). In addition, there was a greater number of males (61%) diagnosed and an overwhelming majority was Caucasian (89%). Our results for the state of Pennsylvania are similar to the data discussed in other studies (1). In addition, most cases (>64%) were localized, but a significant increase in both localized and regional disease was seen. Notably, one-third were advanced at the time of diagnosis, and classified as either regional or distant disease.

Overall, RCC incidence in Pennsylvania had nearly doubled over the past 27 years, from 9.9 patients per 100,000 population in 1990 to 18.0 patients per 100,000 population. This increase was primarily driven by the increase in local and regional defined disease. Fortunately, rates of distant disease remained stable. In addition, while there was an observed increase in disease incidence amongst both African Americans and Caucasians, the annual percentage increase was higher for African Americans at 2.72% when compared to Caucasians at 2.33%, although the difference between both groups did not reach statistical significance. These findings are consistent with other population-based data (5).

Comparatively, rising trends in Pennsylvania was similar to nationwide trends, where incidence of RCC in the United States increased from approximately eight patients per 100,000 population in 1992 to approximately 14 patients per 100,000 population in 2015. Although different states followed similar rising trends, reports showed significant variation of incidence of RCC; Alaska had the highest rates of RCC (21 per 100,000 population), whereas Utah and Hawaii (10 per 100,000 population) has the lowest rates of RCC (14). Further research is needed to evaluate such important geographic variation.

Over the last 2 decades, worldwide incidence of RCC increased from 4.7 to 4.9 per 100,000 population and is projected to increase further (15). Incidence of RCC varied significantly around the globe, where the United States had the highest incidence rate of RCC (11.7 per 100,000 population) followed by Western Europe (9.8 per 100,000 population) and Australia/New Zealand (9.2 per 100,000 population). Africa had the lowest incidence rates at below 0.2 per 100,000 population (16). The United States and the United Kingdom had a rising trend (17, 18), whereas countries such as Sri Lanka, Trinidad and Tobago, and Qatar had a declining trend (15). This discrepancy may be attributed to variation of imaging utilization and exposure to risk factors in certain geographic areas around the globe.

RCC is more commonly diagnosed in the older population (12) with our data showing that 86% of RCC patients in Pennsylvania were aged 50 years or older at the time of diagnosis and the overall incidence is rising. This rising incidence of RCC is likely multifactorial. One plausible explanation could be due to increased incidental diagnoses secondary to increased utilization of cross-sectional imaging. A recent study discovered a significant increase in advanced imaging studies in the work-up of unrelated abdominal symptoms (19). While the study noted that most renal masses found during the study appeared benign, it often led to additional work-up. One study even showed that increasing rates of CT imaging resulted in higher risk of undergoing nephrectomy in certain parts of the United States (20). The authors felt that while higher rates of nephrectomy likely reflected incidental detection of renal masses, a notable downside of excessive CT imaging is additional surgery.

Other factors should also be considered for the increased incidence of RCC. For example, if increased diagnosis of RCC is primarily driven by increased utilization of cross-sectional imaging, then it would be expected to observe a more significant increase in the older population since abdominal imaging is more commonly utilized in the older age group (21). Interestingly, our study showed that the rates of increase was more pronounced in the younger population (<50 years), suggesting that additional factors play a role.

Other risk factors related to RCC are known and continue to evolve and prompts a real change in incidence rates (10). Smoking has long been considered an environmental factor not only for the incidence of disease but also for the risk of distant metastasis and lymph node involvement (22, 23). While smoking in the United States trends downward, chronic medical conditions that increase the risk of RCC have been on the rise. For example, the rates of obesity and diabetes mellitus have increased threefold and sevenfold, respectively, since the 1960s (8, 10, 24, 25). A study by Dobbins et al. evaluated the association between obesity and cancer risk (26). They determined the relative risk of RCC was 1.57 in obese men and 1.72 in obese women when compared to people with a normal BMI. In addition, several studies revealed that diabetes mellitus significantly increased the risk of RCC, independent of other co-morbid conditions such as obesity and hypertension (27, 28). With the prevalence of co-morbid conditions continuing to rise in the United States, their effects are only beginning to be revealed.

Geospatial mapping and county-level data reveal wide-spread disease and relatively well-distributed spread of disease across majority of the counties, regardless of the level of urbanization, which may be attributed in part to the widespread cross-sectional imaging even at small rural healthcare facilities.

This study has several limitations that we identified. The possibility of misclassification or unmeasured changes in stage reporting can be seen in any registry-based observational data. Also, registry observational studies lack standardized and centralized pathologic review. This impedes a uniform diagnostic assessment of the malignancies both in terms of diagnosis and stage. Lastly, selection bias is likely imbedded in the data collection process. However, these data strengthen our understanding of the incidence and geographic distribution of RCC, and how increasing prevalence of risk factors may influence its incidence.

## Conclusions

Incidence of RCC has increased over the past three decades in Pennsylvania. These increases are predominantly driven by a greater diagnosis of localized disease, although one-third of the cases are regional or metastatic at presentation. Higher rates of increase were more pronounced in the younger, male population. Geospatial investigation implicates growing “hot spots” of RCC in certain parts of the state.
